# Supplementary material for: High-fidelity single-frame computational super-resolution using signal-preserving denoising-enabled deconvolution
Source: Nat Commun. 2026 Mar 17;17:4056. doi: 10.1038/s41467-026-70791-8 (PMC13139407; doi:10.1038/s41467-026-70791-8)
Supplement: Supplementary file 1 — Supplementary Information [file 41467_2026_70791_MOESM1_ESM.pdf]

Contents

Supplementary Fig. 1-11
Supplementary Table 1, 2

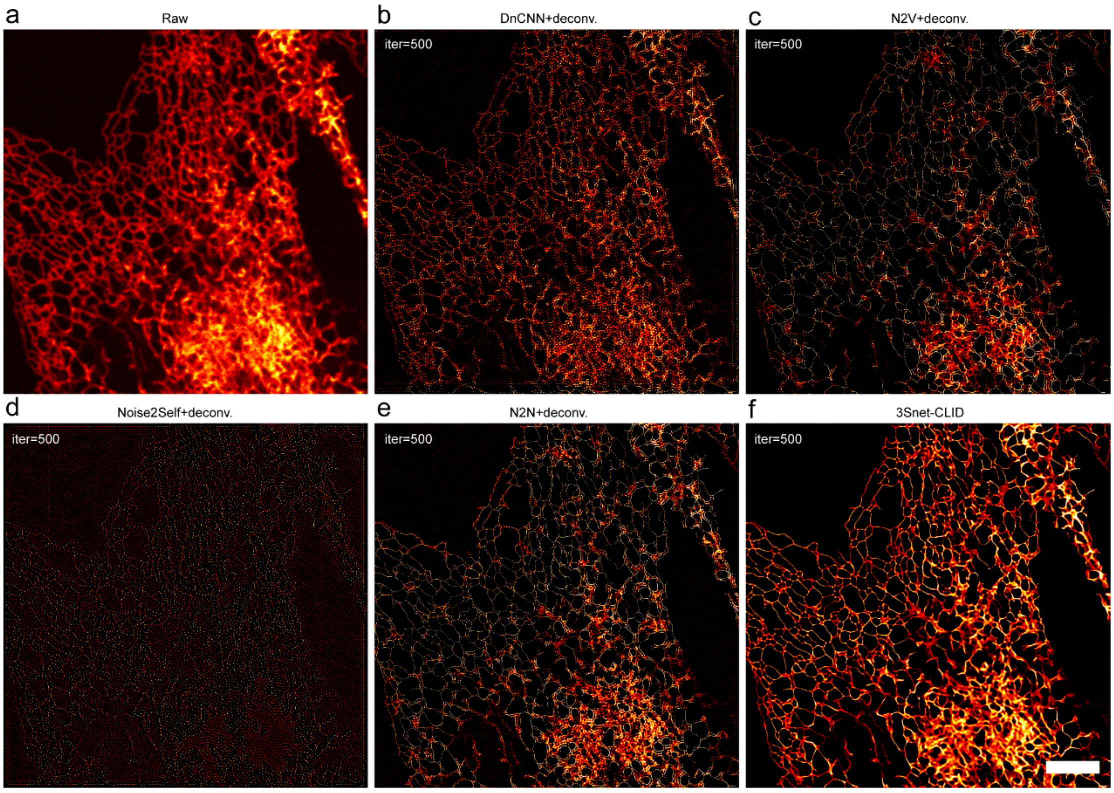

Supplementary Fig. 1 Comparison of 3Snet-CLID with other deep learning denoising methods. The ER structure of U-2 OS cells was labeled with mScarlet3-S2. Single-frame images were denoised using various approaches were processed using Lanczos upsampling by a factor of 3, followed by RL deconvolution with a theoretically derived Bessel PSF, using 500 iterations. Scale bar: 5 µm.

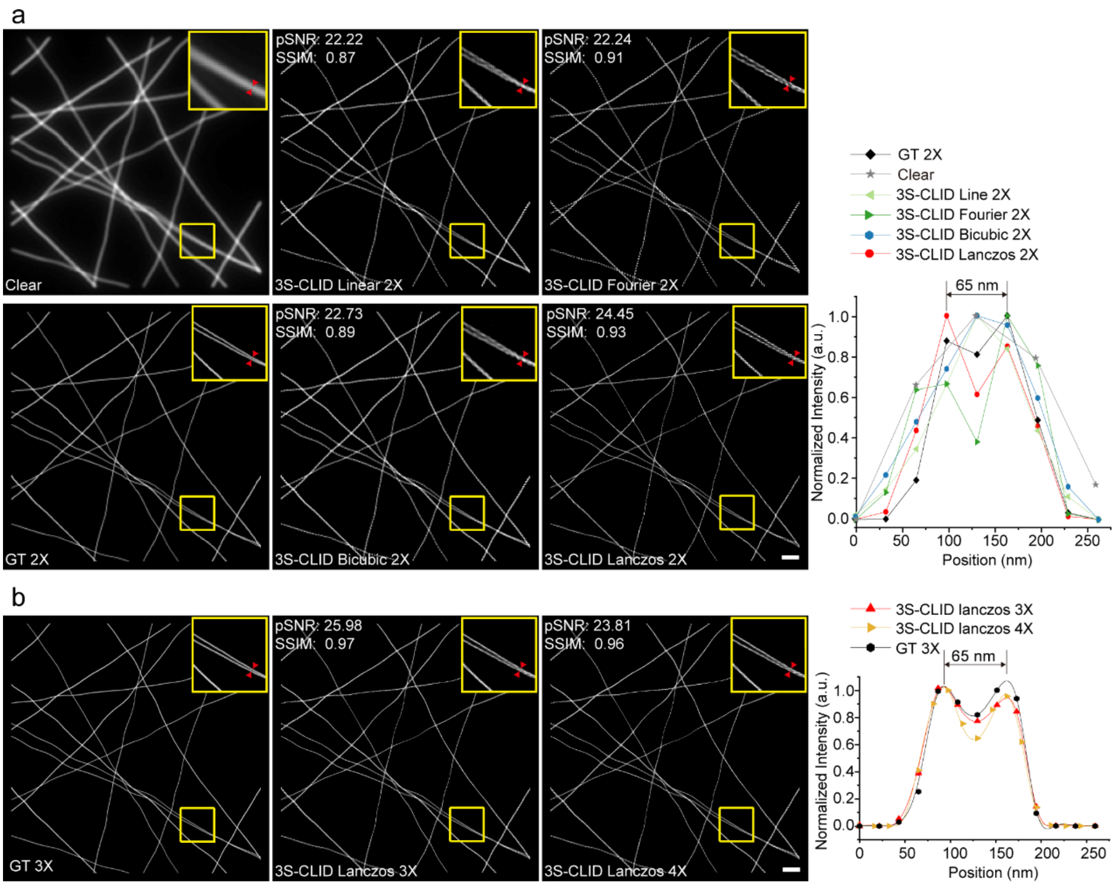

Supplementary Fig. 2 Assessment of upsampling approaches for 3S-CLID. **a**, 3S-denoised synthetic structures were upsampled by Linear, Fourier, Bicubic or Lanczos interporation approache for RL deconvolution. 5% Poisson noise and 5% Gaussian noise were added to the acquired a blank region of a fixed cell under WF illumination, and 3S-denoised images were processed using different upsampling approaches by a factor of 2, followed by RL deconvolution with a theoretically derived Bessel PSF, using 1000 iterations. The pSNR and SSIM of 3S-CLID images were measured to compare the performance of the four upsampling approaches. Scale bar: 1 μm. **b**, 3S-denoised images were processed using Lanczos upsampling approach by different factors, followed by RL deconvolution with a theoretically derived Bessel PSF, using 1000 iterations. Scale bar: 1 μm.

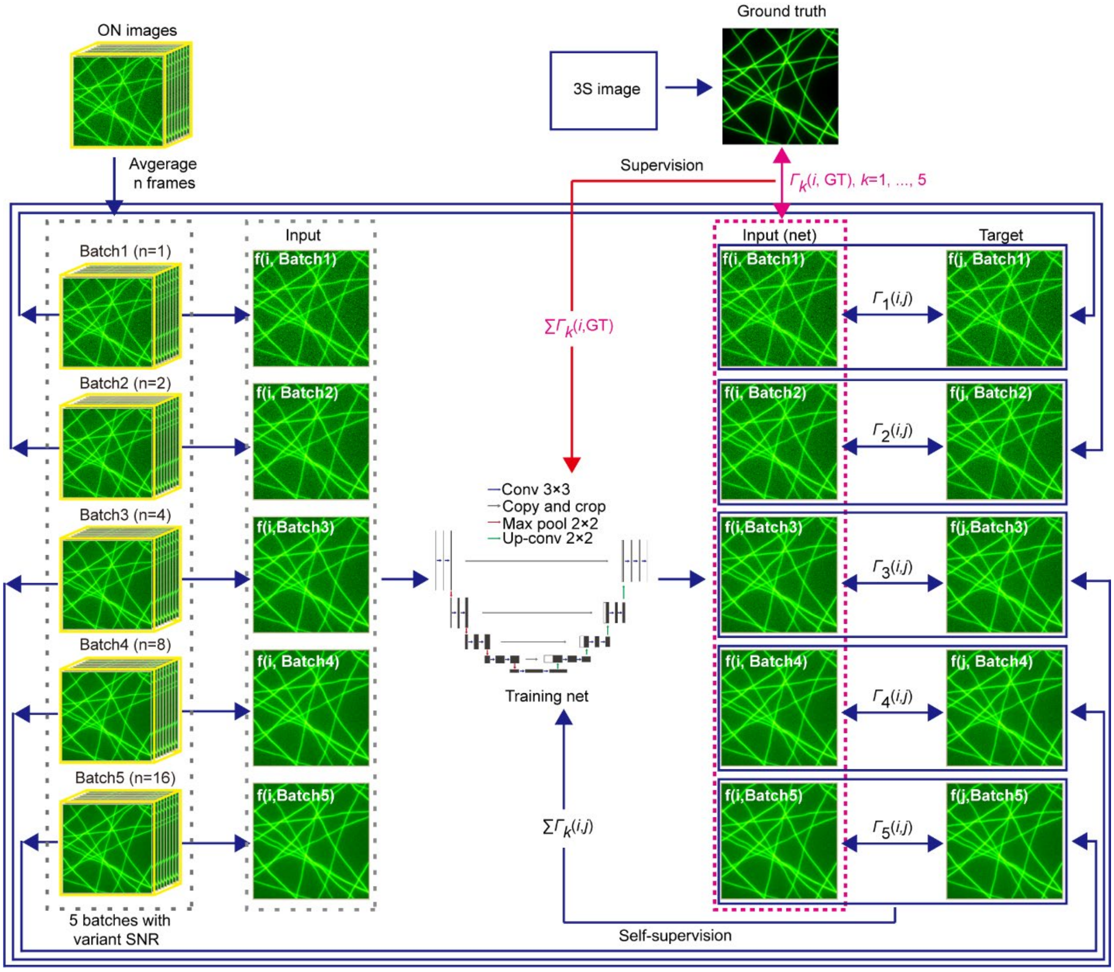

Supplementary Fig. 3 Dataset generation for 3Snet-CLID. To generate dataset for self-supervision, we first extract ON state images from the raw ON/OFF image sequences captured using SkylanS photoswitching. To generate training sets with diverse noise levels, 50 frames of images from the ON state are organized into five datasets using the combination formula C_n^k, where n equals 50 and k is 1, 2, 4, 8 or 16. Next, 50 elements are randomly selected from each dataset and the average of the k images in each chosen element is calculated. Finally, two images in each batch are randomly selected as the input and target data to the U-net model. For supervision, the clear image obtained by 3S-denoising was used as the ground truth.

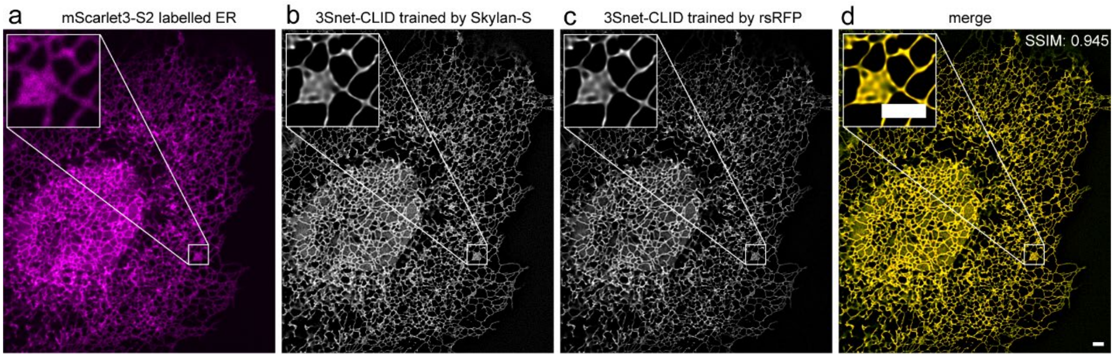

Supplementary Fig. 4 3Snet-CLID trained on Skylan-S can resolve structures labeled with mScarlet3-S2. **a**, Representative single frame image showing the ER in a live U-2 OS cell, labeled with the red FP mScarlet3-S2. **b**, SR image of mScarlet3-S2-labeled ER obtained using Skylan-S-trained 3Snet model. **c**, SR image of mScarlet3-S2-labeled ER obtained using rsRFP-trained 3Snet model. **d**, Merge image of **b** and **c** to indicate that the network trained on green RSFP successfully reconstructed structures labeled with traditional red FP, which is consistent with the results from the network trained on red RSFP. Scale bar: 2 μm.

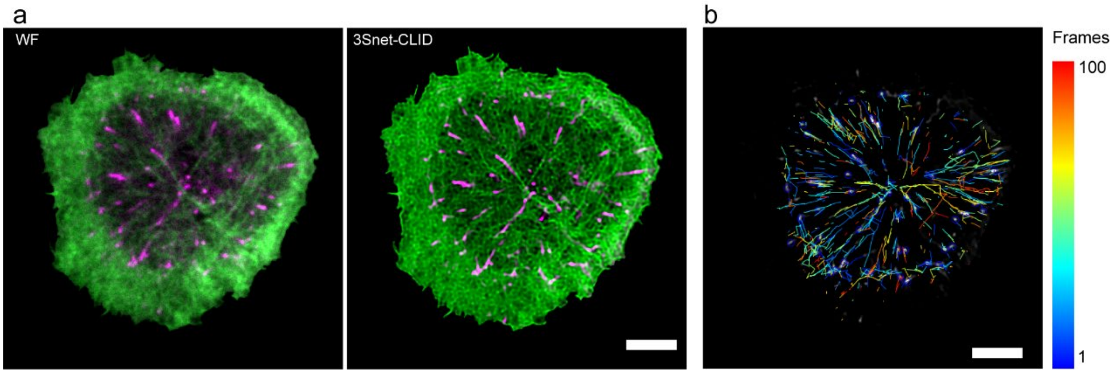

Supplementary Fig. 5 Dual-color imaging of F-tractin-StayGold-labeled F-actin (green) and EB1-mScarlet-I-labeled growing microtubule plus-ends (magenta) in living Jurkat T cells. **a**, A representative example showing a WF microscopy image alongside its reconstruction using the 3Snet-CLID method. Scale bar: 5 μm. **b**, Dynamic EB1 on growing microtubule plus-ends were automatically tracked over time using TrackMate. The color bar represents the frame number when different EB1 trajectories appear. Scale bar: 5 μm.

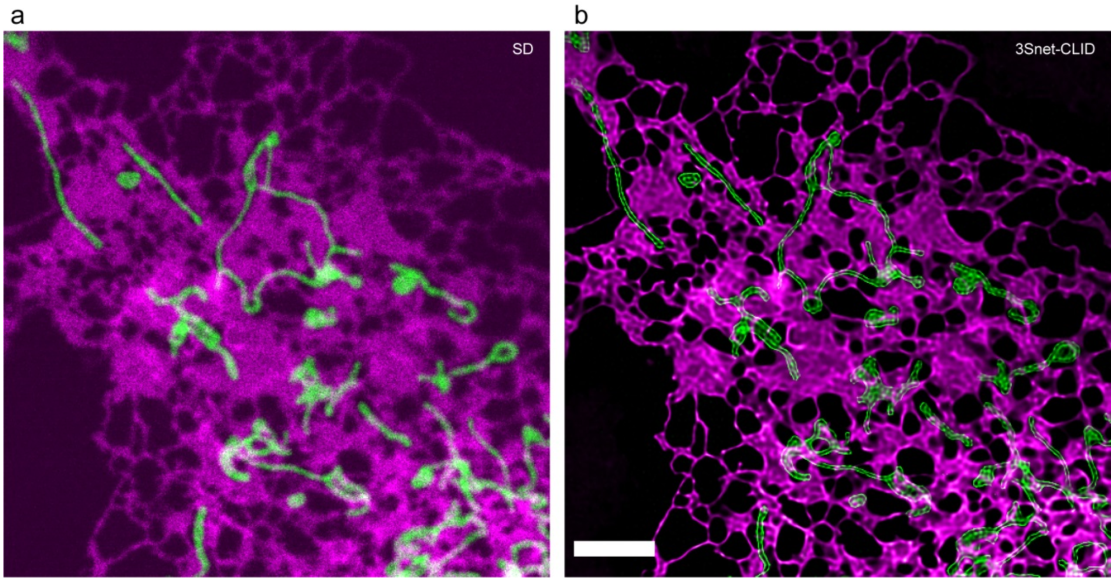

Supplementary Figure 6 Single-frame SR imaging of live COS-7 cells with 3Snet-CLID. The universality of 3SnetCLID was verified by Olympus SpinSR10 microscope. The mitochondrial outer membrane protein Tom20 and the ER protein Sec61β in COS-7 cells were labeled with StayGold and mScarlet-I, respectively. **a** and **b**, representative images of SD (**a**) and 3Snet-CLID (**b**). Scale bar: 2 μm.

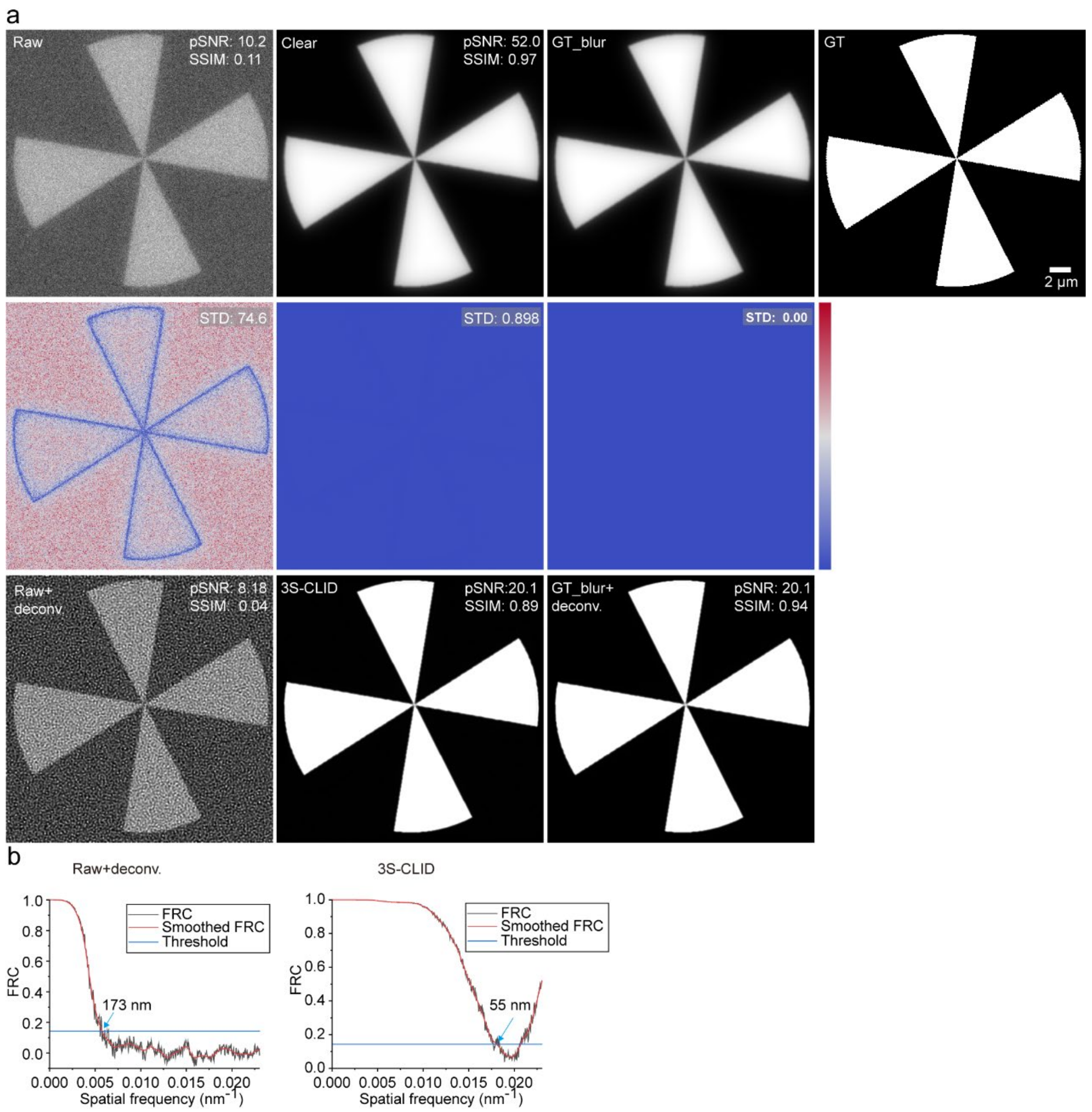

Supplementary Figure 7 Validation of 3S-CLID using synthetic sector test pattern. **a**, Top row: ground-truth (GT) image and the pSNR (peak signal-to-noise ratio) and SSIM (structural similarity) values for the raw (noisy) image and the Clear (3S-denoised) image relative to GT_blur (blurred ground truth). Middle row: Data uncertainty results of top row images indicated by the averaged standard deviation (STD). The color bar blue to red represents the STD value. Bottom row: deconvolution of top row images. The Raw images were created by acquiring a blank region of a fixed cell under WF illumination, and further injection of 5% Poisson noise and 5% Gaussian noise. The resulting images were processed using Lanczos upsampling by a factor of 3, followed by RL deconvolution with a theoretically derived Bessel PSF, using 500 iterations. Scale bar: 2 μm. **b**, FRC analysis of denoised images; the line marks the 1/7 threshold.

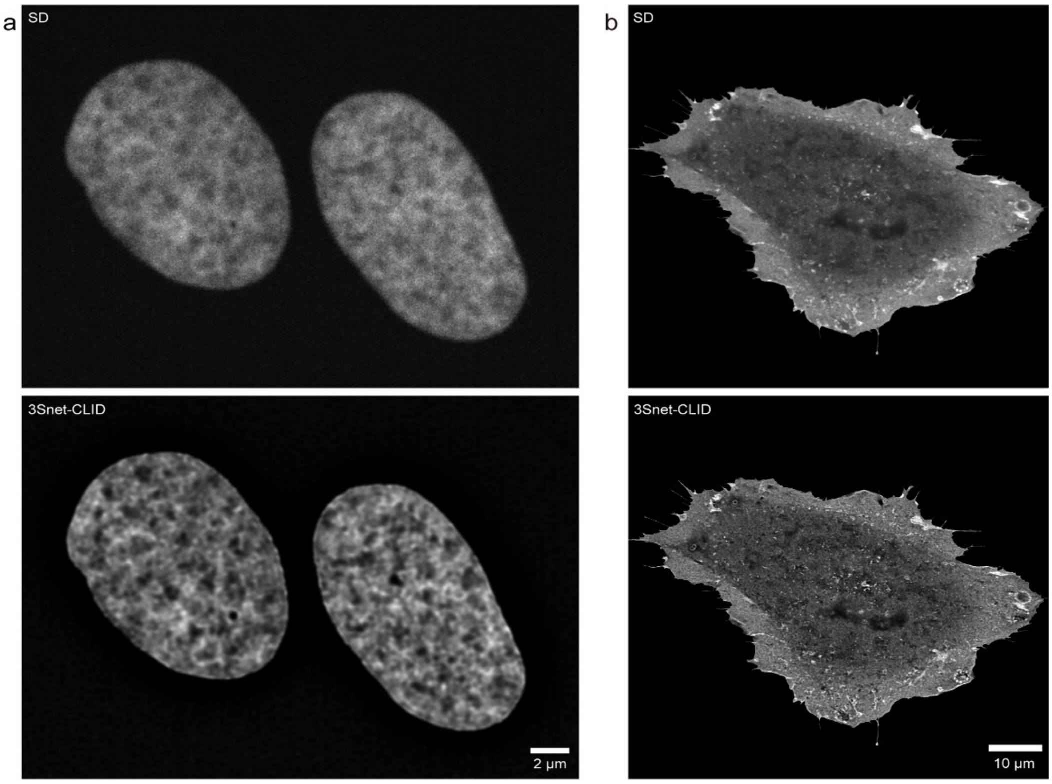

Supplementary Figure 8 SD and 3Snet-CLID images of chromatin or lipid membrane in fixed U-2 OS cells expressing mSG-labeled H2B (**a**) and LactC2 (**b**), respectively, under spinning disc (SD) microscopy. Scale bars: 2 µm (**a**) and 10 µm (**b**).

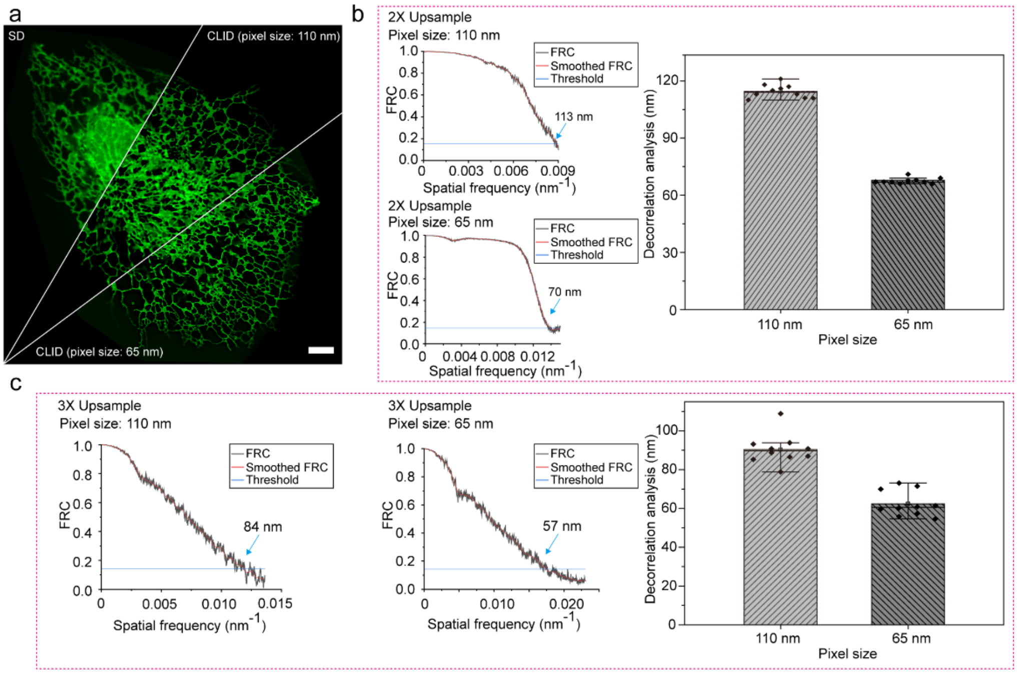

Supplementary Figure 9 Assessment of CLID resolution with cameras of different pixel sizes. **a**, Comparison of SD and CLID-resolved images acquired using cameras with different pixel sizes. Scale bar: 5 µm. **b** and **c**, Fourier ring correlation (FRC) and corresponding decorrelation analysis are used to quantify the resolution of the denoised images by the 2 × upsampling (**b**) and the 3 × upsampling (**c**). For the box-and-whisker plots, the center line represents the median, the box spans the interquartile range (IQR; 25th to 75th percentiles), and whiskers extend to 1.5× IQR. Outliers are plotted as individual points.

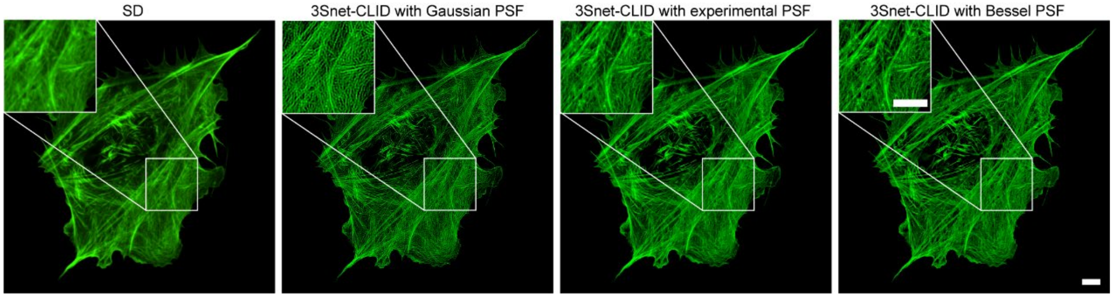

Supplementary Figure 10 Comparison of 3Snet-CLID results with different PSF models. Left to right: SD image, 3Snet-CLID images with Gaussian, experimental or Bessel PSFs. Scale bar: 5 μm.

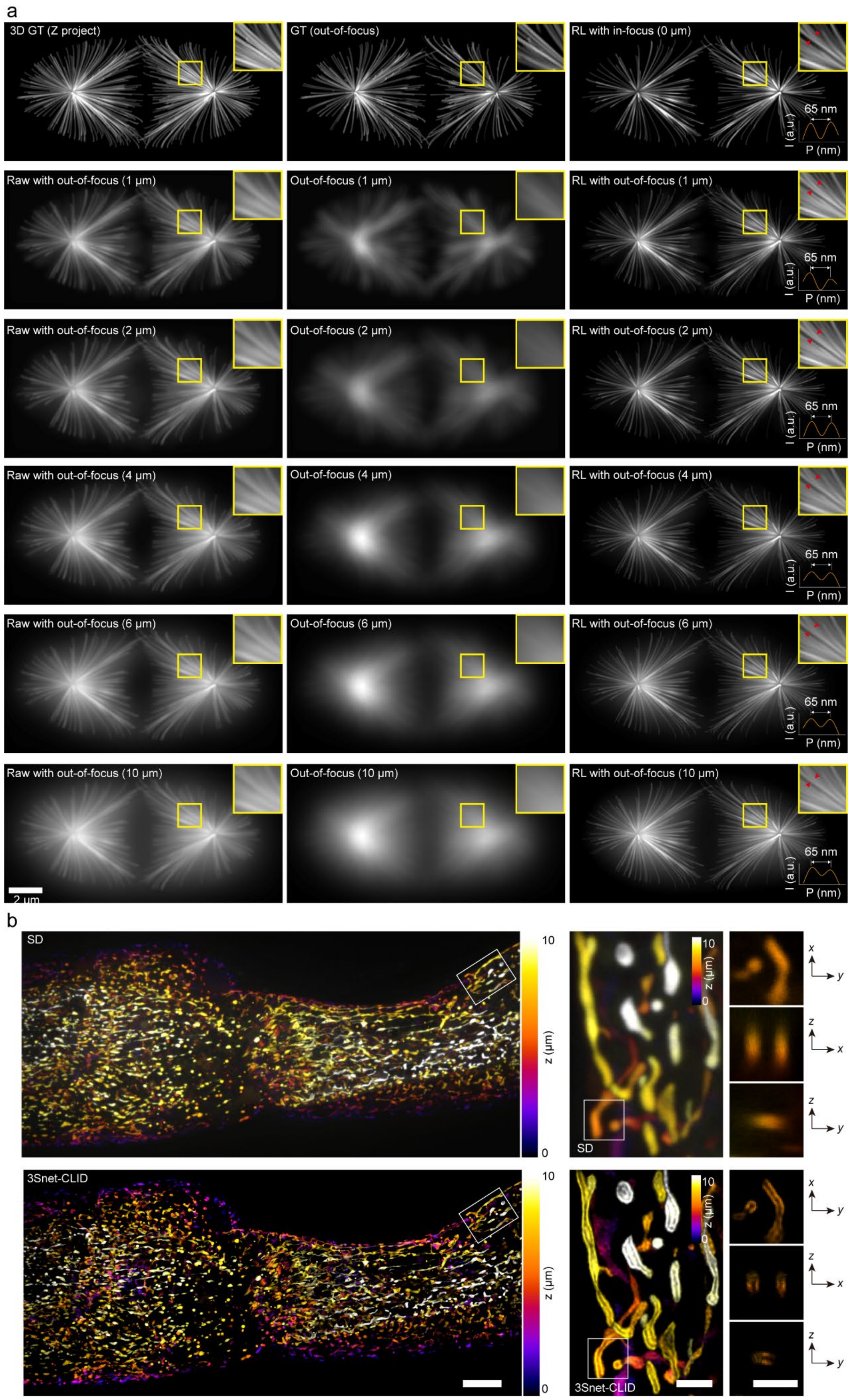
a
3D GT (Z project)
GT (out-of-focus)
RL with in-focus (0 μm)
Raw with out-of-focus (1 μm)
Out-of-focus (1 μm)
RL with out-of-focus (1 μm)
Raw with out-of-focus (2 μm)
Out-of-focus (2 μm)
RL with out-of-focus (2 μm)
Raw with out-of-focus (4 μm)
Out-of-focus (4 μm)
RL with out-of-focus (4 μm)
Raw with out-of-focus (6 μm)
Out-of-focus (6 μm)
RL with out-of-focus (6 μm)
Raw with out-of-focus (10 μm)
Out-of-focus (10 μm)
RL with out-of-focus (10 μm)
65 nm
I (a.u.)
P (nm)
2 μm
b
SD
3Snet-CLID
z (μm)
10
0
x
y
z

Supplementary Figure 11 Impact of out-of-focus blur on 3Snet-CLID performance. **a**, Simulation data analysis. The 3D-PSF was generated by Deconwolf. The simulated data[62] are then converted into an image containing defocus signals using the following formula:

$$F(x,y) = \sum_{-n}^{n} [GT(x,y,\mathrm{n}) \otimes 3D\_PSF(x,y,\mathrm{n})]$$

By varying the *dz* parameter, 3D-PSFs with different degrees of axial blur are generated to capture defocus signals at various depths. When n=0, *GT*(x,y,0) corresponds to the in-focus signal, and *3D_PSF*(x,y,0) represents the 2D-PSF. Defocus depths of 1 µm, 2 µm, 4 µm, 6 µm, and 10 µm are used. Each layer is treated as a 2D defocused image, and the resulting image is then processed using RL deconvolution with the corresponding 2D-PSF. Scale bar: 2 µm. **b**, Performance of 3Snet-CLID at different imaging depths in SD microscopy of *C. elegans* ($P_{hyp\text{-}7}$::TOMM-20::GFP). Scale bar: 10 µm (left), and Scale bar: 2 µm (middle and right).

	Pixels, No. of frames	Running time		Ratio of time (sparse/CLID)
		3Snet-CLID	sparse-deconv.	
Fig.3a	512×512, 1	5 s	20 min	240
Fig.3d	1024×1024, 1	9 s	24 min	160
Fig.4	512×512, 100	1 min 37 s	1 h 20 min	49.5

Supplementary Table 1. Computational efficiency comparison across different imaging configurations.

Processing time comparison between 3Snet-CLID and sparse deconvolution for datasets corresponding to Fig. 3a, Fig. 3d, and Fig. 4. Values indicate 3Snet-CLID runs ~50–240× faster than sparse deconvolution with optimized parameters.

Figures	Microscope	Sample/ Structure	Fluorophore	Laser (nm)	Pixel Size (nm)	Objective lens	RL iteration times	ROI	Time Points	Z-step	Exposure time (ms)	Interval (s)	Total Acquisition time (s)
Fig.1d	WF	Fixed U-2 OS cells/ F-actin	Skylan-S	488	65	100× NA1.49	500	512 × 512	100	——	50	0	5
Fig.1f	Nikon Ti2-W1	Fixed U-2 OS cells/F-actin	Skylan-S	488	65	100× NA1.45	500	1408 × 1408	100	——	50	0	5
Fig.2c	WF	100 nm FluoSpheres™	——	488	65	100× NA1.49	500	252 × 240	1	——	100	0	0.1
		40 nm FluoSpheres™		488	65	100× NA1.49	500	512 × 512	1	——	100	0	0.1
		20 nm FluoSpheres™		488	65	100× NA1.49	500	252 × 240	1	——	100	0	0.1
Fig.2d	WF	ArgoSIM	——	488	65	100× NA1.49	1000	700 × 700	1	——	100	0	0.1
Fig.2e	WF	Origami	FITC 488	488	65	100× NA1.49	1000	96 × 96	1	——	100	0	0.1
Fig.3a	OMX	Fixed HeLa cells/F-actin	Alexa Fluor® 488	488	80	60× NA1.49	500	512 × 512	1	——	30	0	0.03
Fig.3d	OMX	Fixed HeLa cells/F-actin	Alexa Fluor® 488	488	80	60× NA1.49	500	1024 × 1024	1	——	30	0	0.03
		Fixed HeLa cells/PMP70	Alex Fluor® 647	568	80	60× NA1.49	500	1024 × 1024	1	——	200	0	0.2
Fig.4	WF	Live Jurkat T cells/F-tractin	StayGold	488	65	100× NA1.49	500	512 × 512	100	——	50	1	105
Fig.5a	Nikon Ti2-W1	Fixed U-2 OS cells/NUP96	sfGFP	488	65	100× NA1.45	500	289 × 202	1	——	200	0	0.2
Fig.5e	Nikon Ti2-W1	Live U-2 OS cells/ER	mScarlet3-S2	561	65	100× NA1.45	500	1024 × 1024	1000	——	50	0.36	460
		Live U-2 OS cells/mitochondria	mStayGold	488	65	100× NA1.45	500	1024 × 1024	1000	——	50		

SI_Fig.1	Nikon Ti2-W1	Live U-2 OS cells/ER	mScarlet3-S2	561	65	100× NA1.45	500	1024 × 1024	1	——	50	0	0.05
SI_Fig.4	Nikon Ti2-W1	Live U-2 OS cells/ER	mScarlet3-S2	561	65	100× NA1.45	500	1024 × 1024	1	——	50	0	0.05
SI_Fig.5	WF	Live U-2 OS cells/F-tractin	StayGold	488	65	100× NA1.49	500	512 × 512	100	——	50	1	110
		Live U-2 OS cells/EB1	mScarlet-I	561	65	100× NA1.49	500	512 × 512	100	——	50		
SI_Fig.6	Olympus SpinSR10	Live COS-7/ER	mScarlet-I	561	65	100× NA1.45	50	512 × 512	100	——	50	0	5
		Live COS-7/mitochondria	StayGold	488	65	100× NA1.45	50	512 × 512	100	——	50	0	5
SI_Fig.8a	Nikon Ti2-W1	Fixed U-2 OS cells/chromatin	mStayGold	488	65	100× NA1.45	500	323 × 428	1	——	50	0	0.05
SI_Fig.8b	Nikon Ti2-W1	Fixed U-2 OS cells/lipid membrane	mStayGold	488	65	100× NA1.45	500	1192 × 1192	1	——	50	0	0.05
SI_Fig.9	Nikon Ti2-W1	Fixed U-2 OS cells/ER	mStayGold	488	65	100× NA1.45	500	600 × 600	1	——	50	0	0.05
SI_Fig.10	Nikon Ti2-W1	Fixed U-2 OS cells/F-actin	mStayGold	488	65	100× NA1.45	500	1095 × 1095	1	——	50	0	0.05
SI_Fig.10	Nikon Ti2-W1	Live C. elegans/mitochondria	GFP	488	65	100× NA1.45	50	1041 × 2048	——	101	50	0	5.05

Supplementary Table 2. Imaging parameters and sample characteristics for diverse subcellular structures resolved by 3Snet-CLID.

Summary of experimental conditions for the imaging datasets presented in this study, demonstrating that 3Snet-CLID resolves structures spanning micrometer to nanometer scales—including lines, puncta, rings, tubules, filaments, and near homogeneous patterns—without structure specific tuning. The table lists the figure references, microscope types, labeled subcellular structures, fluorophores, laser wavelengths, objective lenses, deconvolution iteration numbers, exposure times, and total acquisition times for each dataset.
