# Supplementary material for: High-fidelity single-frame computational super-resolution using signal-preserving denoising-enabled deconvolution
Source: Nat Commun. 2026 Mar 17;17:4056. doi: 10.1038/s41467-026-70791-8 (PMC13139407; doi:10.1038/s41467-026-70791-8)
Supplement: Supplementary file 2 — Description of Additional Supplementary Files [file 41467_2026_70791_MOESM2_ESM.pdf]

Description of Additional Supplementary Files

Supplementary Movie 1 | Dynamic actin remodeling during immune synapse formation in live Jurkat T cells captured by 3Snet-CLID

Time-lapse imaging of live Jurkat T cells showing actin dynamics during immune synapse formation with single-frame temporal resolution (corresponding to Fig. 4). 3Snet-CLID enables visualization of: (i) constant retrograde flow of lamellipodial actin networks in the distal supramolecular activation cluster (dSMAC); (ii) variable centripetal movement of lamellar actin networks in the peripheral supramolecular activation cluster (pSMAC); (iii) convergence of actin arcs along shared trajectories (white arrowheads); (iv) non-uniform intensity distributions along actin trajectories; (v) dynamic variations in actin cluster sizes at the outer pSMAC edge; and (vi) coalescence of individual actin bundles into arcs at the dSMAC-pSMAC boundary progressing inward (yellow arrows). The enhanced resolution (4.3-fold improvement) facilitates quantitative assessment of actin arc orientation and polarity within the pSMAC region compared to widefield microscopy.

Supplementary Movie 2 | Dual-color 3Snet-CLID imaging of F-actin and microtubule plus-end dynamics during immune synapse formation

Dual-color time-lapse imaging of live Jurkat T cells showing F-actin labeled with F-tractin-StayGold (green) and microtubule plus-ends labeled with EB1-mScarlet-I (magenta), captured using 3Snet-CLID (corresponding to Supplementary Fig. 5). Automated tracking of EB1 comets reveals: (i) radial movement of microtubule plus-ends from the two poles of the microtubule-organizing center (MTOC) towards the cell periphery with polar distribution; (ii) non-overlapping trajectories of EB1 molecules at distinct growing plus-ends over time; (iii) EB1 signals originating from non-MTOC regions; and (iv) differential disappearance of EB1 molecules within the cell body versus the distal dSMAC layer.

Supplementary Movie 3 | Dynamic ER-mitochondria interactions captured by dual-color 3Snet-CLID

Dual-color time-lapse imaging of live cells showing dynamic interactions between the endoplasmic reticulum (ER) and mitochondria at single-frame temporal resolution using 3Snet-CLID (corresponding to Fig. 5). The green channel resolves mitochondrial outer membranes, including stretched thin membrane structures (resolution improved from 363 nm to 68 nm), while the red channel reveals finer tubular ER and grid-like sheet structures (resolution improved from 346 nm to 64 nm). 3Snet-CLID captures dynamic events including: (i) ER-mediated morphological changes in mitochondria through dynamic generation and disappearance of ER tubules; (ii) stretching, recycling, and fusion of ER structures; and (iii) previously unreported lateral fusion events of mitochondrial outer membranes (region 3).

Supplementary Movie 4 | Validation of 3Snet-CLID resolution robustness across axial depths

Z-stack imaging demonstrating 3Snet-CLID super-resolution capability at different axial depths within ~10 µm thick samples. Despite susceptibility to out-of-focus blur inherent to 2D PSF-based algorithms, 3Snet-CLID reliably maintains resolution performance throughout the sample depth in both simulated and spinning disk (SD) microscopy data (corresponding to Supplementary Fig. 11).
